# Supplementary material for: Comparative Analysis of Major Flavonoids among Parts of Lactuca indica during Different Growth Periods
Source: Molecules. 2021 Dec 8;26(24):7445. doi: 10.3390/molecules26247445 (PMC8705863; doi:10.3390/molecules26247445)
Supplement: Supplementary file 1 [file molecules-26-07445-s001.zip › molecules-1496271-supplementary.pdf]

Supplementary Materials: Comparative Analysis of Major Flavonoids among Parts of *Lactuca indica* during Different Growth Periods

Table S1. Dynamic accumulation of six compounds in roots of *Lactuca indica* L. *cv*.Mengzao (μg/g)

Harvest time	Luteolin	Rutin	Quercetin	Luteolin-7-O-glucoside	Apigenin	Kaempferol
Vegetative stage	-	172.094±4.02^{a}	-	4.987±0.12^{a}	1.725±0.13^{a}	0.936±0.04
Budding stage	-	8.607±0.11^{c}	-	0.646±0.05^{e}	-	-
Initial flowering stage	-	2.583±0.22^{d}	-	0.458±0.03^{f}	0.901±0.05^{b}	-
Middle flowering stage	-	23.215±1.34^{b}	-	2.315±0.15^{c}	0.678±0.03^{b}	-
Peak flowering stage	-	7.801±1.61^{c}	-	2.798±0.24^{b}	0.894±0.07^{b}	-
Filling stage	-	1.742±0.14^{d}	-	0.879±0.08^{d}	-	-

The "-" indicates bot detected. Different lowercase letters in the same column indicate the significance of the difference between treatments at the level of 0.05, the same as in the following table

Table S2. Dynamic accumulation of six compounds in stems of *Lactuca indica* L. *cv*.Mengzao (μg/g)

Harvest time	Luteolin	Rutin	Quercetin	Luteolin-7-O-glucoside	Apigenin	Kaempferol
Vegetative stage	5.154±0.31^{e}	50.161±2.15^{e}	8.446±0.12^{c}	10.464±1.52^{d}	2.451±0.02^{f}	0.545±0.08^{e}
Budding stage	4.249±0.36^{e}	23.097±2.45^{f}	11.431±0.44^{a}	1.202±0.04^{f}	3.655±0.15^{e}	1.341±0.09^{d}
Initial flowering stage	8.721±1.55^{d}	646.860±14.75^{a}	11.317±0.10^{a}	42.227±0.94^{a}	4.534±0.08^{d}	2.478±0.10^{c}
Middle flowering stage	12.066±0.08^{c}	81.894±7.36^{d}	11.391±0.02^{a}	5.091±0.35^{e}	5.391±0.26^{c}	2.605±0.23^{c}
Peak flowering stage	16.560±0.30^{b}	346.080±12.46^{b}	11.011±0.41^{b}	32.738±1.12^{c}	9.255±0.22^{a}	3.611±0.10^{b}
Filling stage	20.794±2.58^{a}	233.559±8.76^{c}	11.091±0.65^{b}	34.208±2.02^{b}	5.814±0.58^{b}	4.704±0.58^{a}

Table S3. Dynamic accumulation of six compounds in flowers of *Lactuca indica* L. *cv*.Mengzao (μg/g)

Harvest period	Luteolin	Rutin	Quercetin	Luteolin-7-O-glucoside	Apigenin	Kaempferol
Initial flowering stage	445.518±9.42^{a}	1487.904±1.85^{a}	18.951±0.86^{a}	170.516±9.56^{c}	142.117±4.49^{a}	58.970±0.22^{a}
Middle flowering stage	310.766±4.68^{b}	1376.498±10.38^{b}	17.494±3.46^{b}	211.15±6.71^{b}	134.614±15.73^{b}	35.598±3.70^{b}
Peak flowering stage	186.457±1.83^{c}	1246.715±2.36^{c}	14.143±0.15^{c}	272.810±3.76^{a}	131.769±1.21^{b}	27.886±0.19^{c}
